# Knowledge and attitudes regarding elder abuse in the community, Eastern Province Saudi Arabia

**DOI:** 10.1186/s12877-020-1416-4

**Published:** 2020-03-04

**Authors:** Zahra E. Almakki, Shaher Z. Alshehri, Moataza M. Abdel Wahab

**Affiliations:** 1 Department of Family Medicine, Imam Abdulrahman Bin Faisal University, P.O.Box 1982, Dammam, 31441 Kingdom of Saudi Arabia; 2 Biostatistics Department HIPH, Alexandria University, Alexandria, Egypt

**Keywords:** Geriatric, Elder abuse, Knowledge, Neglect

## Abstract

**Background:**

The population of old people is increasing world-wide. Along with the increase in their population, an increase in the cases of elder abuse is expected. This study identifies the knowledge of elder abuse and attitudes towards it among the adult attendees of Al Qatif primary health care centers (PHCCs) in Saudi Arabia.

**Methods:**

In this cross-sectional study, 430 participants at PHCCs, in Al Qatif, in the kingdom of Saudi Arabia completed a questionnaire concerning their knowledge of elder abuse and their attitudes toward the subjects of such abuse.

**Results:**

A total of 430 subjects participated in the study. The mean age of the respondents was 35.6 years. Participants included both caregivers (*n* = 93) and non-caregivers (*n* = 337). The overwhelming majority of respondents 97% stated that it is their individual responsibility to report elder abuse and neglect if they witness any. Similarly, (91.8%) of the participants agreed that elder abuse and neglect is a criminal act and they have the responsibility to prevent such abuse and neglect. Also, (90.7%) were aware of financial abuse, and (93.5%) believed that using swear words can be considered abuse, as it is not part of their culture. The majority of them (90.5%) agreed that occasional manhandling of the older people is violence/abuse.

**Conclusion:**

Most of the participants regarded abuse and neglect of the older people as a serious problem and considered it their responsibility to intervene when they encountered it. Further efforts are required to explore the factors associated with elder abuse to utilize that knowledge in the development of effective interventions to prevent such abuse.

## Background

The World Health Organization (WHO) and the International Network for the Prevention of elder Abuse have recognized the abuse of older people as a significant global problem [1]. Elder abuse is defined as a single or repeated act, or lack of appropriate action, occurring in any relationship where there is an expectation of trust that causes harm or stress to an older person [1]. The American Psychological Association has divided elder abuse into five types: physical abuse, psychological or emotional abuse, financial abuse or exploitation, sexual abuse and caregiver neglect [2].

The main risk factors for elder abuse are social isolation, functional disability, psychological disorder or character pathology, cognitive impairment, and caregiver burnout and frustration [2]. Elder abuse has been associated with a number of negative consequences such as reduced quality of life, negative health outcomes, suicidality and a greater risk of mortality [3].

Globally, the number of persons aged 60 years or above is expected to almost triple within the next few decades, from 672 million in 2005 to nearly 1.9 billion by 2050 [2]. The prevalence of elder abuse varies widely, with reported incidence ranging from (2.2%) in Ireland to (79.7%) in Peru [4].

In the eastern Mediterranean region, elder abuse prevalence has been calculated at (43.7%) in Egypt and (14.7%) in Iran [4]. There appears to be a substantial relationship between the incidence of elder abuse and the culture or religion of a country, an issue which needs further study.

There is also a difference in the attitudes towards the elder abuse even between the people in the health care community and the public community.

A study was conducted in the United States among the primary health care physicians, to assess their knowledge and attitudes regarding the elder abuse and neglect. The study was done in Ohio between large urban, suburban, rural practice setting. The results of the study showed physician need more education about elder abuse [5].

In 2013, a study in Turkey was conducted among volunteer public non-health staff and tradesmen, even though results show that participants had a quite sensitive and positive attitudes toward elder abuse and neglect they still need further education [6]. This shows that it’s very important to address carefully the elder abuse setting in both health care field and public community.

It is important to address the abuse, mistreatment, abandonment and negligence of older people, especially, in a community and culture where older people are dependent on care from family member.

Our study was conducted in al Qatif city, which is a governorate and urban area located in the eastern province Saudi Arabia. The population around more than 500,000 [7]. It is known that most of the old people in this region live within a large family due to Islamic culture or conventional norms.

In Saudi Arabia, there is a limited number of studies addressing geriatric field. Understanding the attitude toward the older people and its predictor of sociodemographic character will help to determine the social need for educational program.

### Aims

To assess knowledge and attitudes regarding elder abuse of the Saudi adult population.

## Methods

### Study design

The study design is cross sectional.

### Study location

The study was conducted at 14 PHCCs in Al Qatif, Eastern province, Kingdom of Saudi Arabia. The centers are divided into three categories according to their number of visitors: more than 200 visitors per day (Category A), 150 to 200 visitors per day (Category B), and fewer than 150 visitors per day (Category C).

### Study population

The study population consisted of attendees between 18 and 59 years old at the PHCCS in Al Qatif.

### Sample size and techniques

Assuming (50%) negative and positive attitudes towards the older people and a (50/50) split between those with good versus not very good knowledge of elder abuse, at (95%) confidence interval level, and accepting a (5%) error rate, the minimum required sample size was calculated to be 384 using Epiinfo.7.0. We decided on a sample size of 430.

$$ Sample\ Size=\frac{Z^2 pq}{\delta^2}=430 $$

*Z* represents the confidence level, with (95%) confidence being the standard choice for this level.

Z = 1.96

*P* (the expected prevalence) = 0.5

*q*=1-p= 1-0.69 = 0.30

*δ*: 0.05 is the error tolerated in the estimation.

The sample was allocated using stratified random sampling of patients in categories A, B and C centers. The sample was chosen by proportional allocation and then by equal allocation within the center (30–40 patient) using a consecutive sampling technique until we had the number needed per center.

### The research tools

The questionnaire was distributed to an equal number of men and women who visited a PHCCs in Al Qatif. There were two prerequisites for participation in the survey. The first one was being from 18 to 59 years of age, and the second one was the ability to communicate in Arabic language. The questionnaire had five sections. The first section included sociodemographic questions. The second section assessed whether people blamed the older people for their abuse (7 items). The third section assessed awareness of different types of abuse of the older people (14 items). The fourth section examined attitude regarding social responsibility related to elder abuse (4 items). Finally, the fifth section assessed knowledge regarding possible causes of elder abuse (10 items).

Questions were answered using a 5-point Likert scale with 5 indicating strongly agree and 1 indicating strongly disagree. Questions indicating a negative attitude were reverse-scored. The attitude scores were calculated as a percentage of the maximum score of 5.

The categories were further reduced into 3 categories, agree (strongly agree and agree), neutral and disagree (strongly disagree and disagree).

Education was categorized into higher education (university and above) and average education (below university level).

### Validity and reliability

The questionnaire was constructed by Muthuvenkatachalam Srinivasan, and was modified and translated into Arabic [8]. Face validity was tested by three consultants in geriatric and family medicine, and the questionnaire was reviewed by an Arabic language expert. A pilot study of 20 subjects was conducted, but is not included in the analysis of the results presented here. The reliability of the questionnaire was acceptable (Cronbach’s alpha =0.758).

### Study variables

Dependent variables pertained to knowledge and practice of elder abuse and neglect.

Independent variables consisted of sociodemographic data (age, gender, education level, nationality, income, marital status, occupation, caregiver, source of income, living, type of family).

### Data collection and management

The data collection began on 16/4/2018 and was completed on 28/5/2018. The data were analysed using statistical product and service solutions (SPSS) 20.0. level of significance was set at 0.05. The descriptive statistics presented here include frequency, percentage, mean and standard deviation. Associations between categorical variables were tested using chi-square tests, and the difference in attitude scores between categories was tested using t-tests. Multiple linear regression was used to identify independent significant factors related to the attitude scores.

### Ethical considerations

To maintain confidentiality, data were collected anonymously with approval of the Research Ethical Committee of the King Fahad medical city. The research did not include any interventional therapies or any form of medical testing. The participants were informed that their participation in the study was voluntary, and that they were free to refuse to participate, or withdraw at any stage without being asked for a reason or persuaded to continue. The participants were informed that their refusal to participate in or withdrawal from the study would have no consequences on their health care and that their information would remain confidential.

## Result

The study consisted of 430 participants from 14 PHCCs in Al Qatif. The participants were equally allocated from both sexes.

The majority of the participants were non-caregivers (78.4%). The percentage of caregivers was (21.6%). Among the caregivers, (51.6%) were caring for their mothers, (43%) were caring for their fathers, and (4.3%) were caring for their father in law (Table 1).

**Table 1:** Sociodemographic characteristics of study participants (*N* = 430)

		caregiver					
		yes (n = 93, 21.6%)		no (*n* = 337, 78.4%)		Total	
		No.	%	No.	%	No.	%
age	< 30 yrs	22	23.7	125	37.1	147	34.2
	30+ yrs	71	76.3	212	62.9	283	65.8
	Min-max	18–58		18–59		18–59	
	mean ± Sd	37.4 ± 10		35.1 ± 10.5		35.6 ± 10.4	
	Median	36		34		35	
gender	male	47	50.5	168	49.9	215	50.0
	female	46	49.5	169	50.1	215	50.0
marital state	single	22	23.7	86	25.5	108	25.1
	married	54	58.1	197	58.5	251	58.4
	divorce	12	12.9	48	14.2	60	14.0
	widowed	5	5.4	6	1.8	11	2.6
education categories	average education	39	41.9	138	40.9	177	41.2
	higher education	54	58.1	199	59.1	253	58.8
education	elementary	0	0.0	1	0.3	1	0.2
	intermediate	4	4.3	10	3.0	14	3.3
	high school	35	37.6	127	37.7	162	37.7
	university	49	52.7	185	54.9	234	54.4
	above university	5	5.4	14	4.2	19	4.4
occupation	government	35	37.6	129	38.3	164	38.1
	special	34	36.6	122	36.2	156	36.3
	retired	8	8.6	25	7.4	33	7.7
	house wife	13	14.0	59	17.5	72	16.7
	student	3	3.2	2	0.6	5	1.2
income	< 5000 SR	48	51.6	218	64.7	266	61.9
	< 5000–10,000 SR	27	29.0	80	23.7	107	24.9
	< 10,000-15000SR	12	12.9	26	7.7	38	8.8
	> 15,000 SR	6	6.5	13	3.9	19	4.4
Source of income	Personal salary	69	74.2	251	74.5	320	74.4
	retired	8	8.6	25	7.4	33	7.7
	special	16	17.2	61	18.1	77	17.9
living	large family	55	59.1	130	38.6	185	43.0
	nuclear family	38	40.9	207	61.4	245	57.0
relative	father	40	43.0			40	43.0
	mother	48	51.6			48	51.6
	sister	1	1.1			1	1.1
	father in law	4	4.3			4	4.3
disability categories	No disability	28	30.1			28	30.1
	disability	65	69.9			65	69.9
disability	physical disability	23	24.7			23	24.7
	mental disability	9	9.7			9	9.7
	Visual impairment	14	15.1			14	15.1
	hearing impairment	11	11.8			11	11.8
	psychiatric disease	8	8.6			8	8.6
	no disability	28	30.1			28	30.1

Participants’ ages ranged from 18 to 59 with an average of 35.6 years. Among participants who are less than 30 years old, (37.1%) were non-caregivers, and (23.7%) were caregivers. While for participants over 30 years old, (76.3%) were caregivers and (62.9%) were non- care givers (Table 1).

About (58.4%) of the sample were married, (25.1%) were single, (14%) were divorced, and (2.6%) were widowed (Table 1).

Most of the participants (58.8%) had university or above education. The remaining percentage (41.2%) had high school or less education (Table 1).

Among all participants in the study sample, (38.1%) were government employees, (36.3%) were private employee, (16.7%) were not employed, (7.7%) were retired, and (1.2%) were students (Table 1).

Among non-caregivers, (64.7%) had monthly incomes that were less than 5000 SR. While among caregivers, (51.6%) had monthly incomes less than 5000 SR. The individuals with a monthly income higher than 15,000 SR were only (4.4%) of all the participants. Almost (74.4%) of participants’ incomes were from salaries, (17.9%) were from investments, and (7.7%) were from retirement earnings (Table 1).

Just over half of the participants (57%) lived in nuclear families, while (43%) lived with extended families (Table 1).

The relative being cared for were disabled in (69.9%) of the cases, and had no disability in (30.1%) of them. Among the disabled, (24.7%) had physical disabilities, (15.1%) had visual impairments, (11.8%) had hearing impairments, (9.7%) had mental disability, and (8.6%) had psychiatric disabilities (Table 1).

The vast majority of the caregivers, (85–86%), disagreed that nagging, complaining and demanding by the older people were legitimate causes of violent behaviours towards the older people.

(75.1%) of the adults believed that the older people would be less exposed to violence if they were more understanding of younger adults’ and children’s problems (Table 2).

**Table 2:** Percentages who blamed the older people for their abuse

		age		gender		education		living		caregiver		disability		Total	
		< 30 yrs	30+ yrs	male	female	average	higher	large family	nuclear family	yes	no	No disability	disability	No.	%
v1 older people often complain and nag, which leads to abusive behaviour	Agree	8.8	13.1	12.1	11.2	15.3	9.1_b_	16.2a	8.2_b_	14.0	11.0	14.3	13.8	50	11.6
	Disagree	89.1	83.7	83.7	87.4	81.4	88.5_b_	81.1a	89.0_b_	83.9	86.1	85.7	83.1	368	85.6
	neutral	2.0	3.2	4.2	1.4	3.4	2.4	2.7	2.9	2.2	3.0	0.0	3.1	12	2.8
v2 older people are demanding, which leads to violent behaviour of the people in their household	Agree	7.5	11.3	10.2	9.8	15.3a	6.3_b_	14.1a	6.9_b_	10.8	9.8	14.3	9.2	43	10.0
	Disagree	91.2	84.5	86.0	87.4	80.8a	90.9_b_	83.2	89.4	83.9	87.5	82.1	84.6	373	86.7
	neutral	1.4	4.2	3.7	2.8	4.0	2.8	2.7	3.7	5.4	2.7	3.6	6.2	14	3.3
v3older people less expose to violence if they understand the problem of their children	Agree	70.7	77.4	74.0	76.3	72.9	76.7	74.1	75.9	69.9	76.6	64.3	72.3	323	75.1
	Disagree	25.9	18.0	20.9	20.5	23.2	19.0	21.6	20.0	25.8	19.3	35.7	21.5	89	20.7
	neutral	3.4	4.6	5.1	3.3	4.0	4.3	4.3	4.1	4.3	4.2	0.0	6.2	18	4.2
v4 If the older people and the young lived separately, there would be no violence.	Agree	12.2	13.1	10.7	14.9	18.6a	8.7_b_	12.4	13.1	12.9	12.8	14.3	12.3	55	12.8
	Disagree	78.2	76.7	79.1	75.3	70.6a	81.8_b_	75.7	78.4	80.6	76.3	82.1	80.0	332	77.2
	neutral	9.5	10.2	10.2	9.8	10.7	9.5	11.9	8.6	6.5	11.0	3.6	7.7	43	10.0
v5 If the older people lived in a nursing home with their peers, less violence would occur.	Agree	12.2	18.0	14.9	17.2	22.6a	11.5_b_	18.4	14.3	19.4	15.1	21.4	18.5	69	16.0
	Disagree	80.3	73.9	78.1	74.0	70.1a	80.2_b_	73.5	78.0	76.3	76.0	78.6	75.4	327	76.0
	neutral	7.5	8.1	7.0	8.8	7.3	8.3	8.1	7.8	4.3	8.9	0.0	6.2	34	7.9
Attitude of blaming	Min-max	32–100	40–100	40–100	32–100	32–100	36–100	40–100	32–100	40–100	32–100	56–100	40–100	32–100	
	Mean	80.68	79.75	80.45	79.68	77.38a	81.94_b_	79.31	80.64	78.58	80.47	76.57	79.45	80.1	
	Median	84	80	84	84	80	84	84	84	80	84	80	84		
	SD	14.23	14.38	14.34	14.32	15.41	13.21	15.08	13.72	14.03	14.39	12.68	14.58	14.3	
v20 I think I have a responsibility to prevent older people abuse and neglect	Agree	96.6	98.6	99.1	96.7	98.3	97.6	98.4	97.6	98.9	97.6	100.0	98.5	421	97.9
	Disagree	1.4	1.4	0.9	1.9	1.1	1.6	1.6	1.2	1.1	1.5		1.5	6	1.4
	neutral	2.0			1.4	0.6	0.8		1.2		0.9			3	0.7
v21 older people abuse and neglect is a criminal act	Agree	97.3	98.6	98.6	97.7	98.3	98.0	98.4	98.0	98.9	97.9	100.0	98.5	422	98.1
	Disagree	2.0	1.1	1.4	1.4	1.1	1.6	1.6	1.2	1.1	1.5		1.5	6	1.4
	neutral	0.7	0.4		0.9	0.6	0.4		0.8		0.6			2	0.5
v22 When I witness older people abuse and neglect, reporting is my individual responsibility	Agree	95.9a	99.3_b_	97.7	98.6	97.7	98.4	97.8	98.4	98.9	97.9	96.4	100.0	422	98.1
	Disagree	2.0	0.4	1.4	0.5	0.6	1.2	1.6	0.4	1.1	0.9	3.6		4	0.9
	neutral	2.0	0.4	0.9	0.9	1.7	0.4	0.5	1.2		1.2			4	0.9
Attitude responsibility	Min-max	40–100	53.3–100	53.3–100	40–100	53.3–100	40–100	53.3–100	40–100	73.3–100	40–100	73.3–100	73.3–100	40–100	
	Mean	91.84	91.87	91.94	91.78	91.30	92.25	91.93	91.81	93.48	91.41	95.71	92.51	91.8	
	Median	100	100	100	100	93	100	100	100	100	100	100	100		
	SD	11.19	9.45	9.73	10.42	9.45	10.48	9.81	10.27	8.85	10.35	7.53	9.24	10.1	

a and b are significantly different at *p* < .05 in the two-sided test of equality for column means. Otherwise no significant difference is present Tests are adjusted for all pairwise comparisons within a row using the Bonferroni correction

Between (76%) and (77%) of the participants disagreed with the claim that if the older people lived separately, or lived in a nursing home with their peers, less violence would occur. The percentage of participants who blamed the older people for abuse was significant. This percentage was around (80.1%) of participants with university and above education, and (80%) of the participants who were living in nuclear families (Table 2).

(98.1%) of participants agreed that individuals had a responsibility to report elder abuse and neglect that they observed. The percentage was even higher (99.3%) among those over age 30 (Table 2).

Demographic data (age, gender, marital status, education level, income, type of family and whether they are caregivers) were entered into regression models as predictors of blaming the older people for their abuse; the only statistically significant factors (F = 6, *p* < 0.0001, adj R^2^ = 6.6%) were higher educational level (B = 4.9), lower income (in reference to income above 15,000SAR, < 10,000-15000SR B = 9.6, < 5000–10,000 SR B = 10.9 and < 5000 SR B = 13.15) and in reference to singles, being married (B = 3.88) or being widowed or divorced (B = 5. 2). However, none of the above variables were significant factors as regards attitudes toward the social responsibility to address elder abuse nor knowledge (This is not shown in the tables).

Table 3 shows the result of the survey regarding different types of elder abuse. Of the surveyed participants, (86.7%) agreed that physically touching of the older people without their consent is sexual abuse, (90.7%) of adults agreed that borrowing money from elderly parents and not returning it is a financial abuse,(96.7%) agreed that abandoning the older people was neglect, (93.5%) agreed that swearing at or in the presence of the older people is abuse, (90.5%) agreed that occasional manhandling of the older people is a type of abuse, (55.1%) agreed that placement of the older people in a nursing home is a type of neglect.

**Table 3:** Awareness about different types of abuse

		age		gender		education		Living		caregiver		disability		Total	
		< 30 yrs	30+ yrs	male	female	average	higher	large family	nuclear family	yes	no	No disability	disability	No.	%
v6 Borrowing money from older people parents and not returning it is not violence	Agree	5.4	5.3	6.5	4.2	7.3	4.0	7.6	3.7	7.5	4.7	7.1	7.7	23	5.3
	Disagree	91.2	90.5	89.3	92.1	87.6	92.9	88.1	92.7	89.2	91.1	92.9	87.7	390	90.7
	neutral	3.4	4.2	4.2	3.7	5.1	3.2	4.3	3.7	3.2	4.2		4.6	17	4.0
v7 Using swear words cannot be considered older people abuse because it is part of my culture	Agree	2.7	2.5	2.8	2.3	2.8	2.4	5.4a	0.4_b_	2.2	2.7		3.1	11	2.6
	Disagree	91.2	94.7	93.0	94.0	92.7	94.1	90.3a	95.9_b_	93.5	93.5	96.4	92.3	402	93.5
	neutral	6.1	2.8	4.2	3.7	4.5	3.6	4.3	3.7	4.3	3.9	3.6	4.6	17	4.0
v8 Manhandling of older people parents/in-law is not violence/abuse	Agree	1.4	3.2	2.8	2.3	3.4	2.0	3.2	2.0	3.2	2.4	7.1	1.5	11	2.6
	Disagree	87.8	91.9	89.3	91.6	91.5	89.7	89.2	91.4	88.2	91.1	89.3	87.7	389	90.5
	neutral	10.9a	4.9_b_	7.9	6.0	5.1	8.3	7.6	6.5	8.6	6.5	3.6	10.8	30	7.0
v9 Physically touching older people individuals without their consent is not a sexual abuse	Agree	2.0	4.9	4.7	3.3	4.5	3.6	4.3	3.7	3.2	4.2	3.6	3.1	17	4.0
	Disagree	83.0	88.7	86.5	87.0	88.1	85.8	85.9	87.3	83.9	87.5	89.3	81.5	373	86.7
	neutral	15.0a	6.4_b_	8.8	9.8	7.3	10.7	9.7	9.0	12.9	8.3	7.1	15.4	40	9.3
v10 Abandonment of the older people is neglect	Agree	95.9	97.2	98.1	95.3	96.0	97.2	97.8	95.9	94.6	97.3	92.9	95.4	416	96.7
	Disagree	3.4	2.8	1.9	4.2	4.0	2.4	2.2	3.7	5.4	2.4	7.1	4.6	13	3.0
	neutral	0.7	0.0	0.0	0.5	0.0	0.4	0.0	0.4		0.3			1	0.2
v11 Placement of the older people in a nursing home is neglect	Agree	48.3a	58.7_b_	55.8	54.4	55.9	54.5	55.1	55.1	61.3	53.4	82.1a	52.3_b_	237	55.1
	Disagree	33.3a	23.7_b_	25.6	28.4	27.1	26.9	25.9	27.8	30.1	26.1	17.9	35.4	116	27.0
	neutral	18.4	17.7	18.6	17.2	16.9	18.6	18.9	17.1	8.6a	20.5_b_	0.0	12.3	77	17.9
v12 It is neglect if an older people, hygiene, nutrition and safety requirements aren’t met.	Agree	91.8	92.9	95.3a	89.8_b_	90.4	94.1	94.6	91.0	92.5	92.6	85.7	95.4	398	92.6
	Disagree	4.1	3.2	2.3	4.7	5.6a	2.0_b_	2.2	4.5	5.4	3.0	10.7	3.1	15	3.5
	neutral	4.1	3.9	2.3	5.6	4.0	4.0	3.2	4.5	2.2	4.5	3.6	1.5	17	4.0
v13 If an older people can’t meet his/her own hygiene, nutrition and housing requirements, he/she is responsible for that self-neglect	Agree	11.6	14.8	11.6	15.8	17.5	11.1	12.4	14.7	15.1	13.4	25.0	10.8	59	13.7
	Disagree	75.5	76.0	77.7	74.0	71.2	79.1	77.3	74.7	74.2	76.3	60.7	80.0	326	75.8
	neutral	12.9	9.2	10.7	10.2	11.3	9.9	10.3	10.6	10.8	10.4	14.3	9.2	45	10.5
v14 İt is neglect if the older people health needs aren’t met or there is a delay in meeting them	Agree	42.2	43.8	41.9	44.7	47.5	40.3	43.8	42.9	44.1	43.0	64.3a	35.4_b_	186	43.3
	Disagree	22.4	25.1	23.3	25.1	23.2	24.9	24.9	23.7	29.0	22.8	21.4	32.3	104	24.2
	neutral	35.4	31.1	34.9	30.2	29.4	34.8	31.4	33.5	26.9	34.1	14.3	32.3	140	32.6
v15 İt is neglect if an older people lives in a home which has unsuitable conditions	Agree	42.2	44.2	42.3	44.7	46.3	41.5	43.8	43.3	45.2	43.0	60.7	38.5_b_	187	43.5
	Disagree	22.4	25.1	23.3	25.1	24.3	24.1	25.9	22.9	29.0	22.8	25.0	30.8	104	24.2
	neutral	35.4	30.7	34.4	30.2	29.4	34.4	30.3	33.9	25.8	34.1	14.3	30.8	139	32.3
v16 İt is abuse if an older people is exposed to violence such as beating, slapping, kicking, biting and throwing things at them	Agree	99.3	99.3	99.5	99.1	98.9	99.6	99.5	99.2	98.9	99.4	100.0	98.5	427	99.3
	Disagree	0.7	0.7	0.5	0.9	1.1	0.4	0.5	0.8	1.1	0.6		1.5	3	0.7
v17 İt is abuse if an older people is exposed to shouting, insults and ridicule	Agree	98.6	99.6	99.5	99.1	99.4	99.2	99.5	99.2	98.9	99.4	100.0	98.5	427	99.3
	Disagree	1.4	0.4	0.5	0.9	0.6	0.8	0.5	0.8	1.1	0.6		1.5	3	0.7
v18 İt is abuse if an older people money and goods are stolen, whether by force or deception	Agree	99.3	100.0	100.0	99.5	100.0	99.6	100.0	99.6	100.0	99.7	100.0	100.0	429	99.8
	Disagree	0.7			0.5		0.4	0.0	0.4		0.3		0.0	1	0.2
v19 İt is abuse to ignore older people, imprison them in their room/home, and exclude them from society.	Agree	93.9	98.2_b_	98.6	94.9_b_	94.9	98.0	96.2	97.1	96.8	96.7	96.4	96.9	416	96.7
	Disagree	2.7	0.7	0.9	1.9	1.7	1.2	2.2	0.8	1.1	1.5	3.6		6	1.4
	neutral	3.4	1.1	0.5a	3.3_b_	3.4a	0.8_b_	1.6	2.0	2.2	1.8	0.0	3.1	8	1.9

a and b are significantly different at *p* < .05 in the two-sided test of equality for column means. Otherwise no significant difference is present Tests are adjusted for all pairwise comparisons within a row using the Bonferroni correction

The majority of participants (92.6%) agreed that not meeting the requirements of the older individual’s hygiene, nutrition or safety is a form of neglect. Also a majority of the participants (75.8%) disagreed with the statement that if an older individual was unable to meet his/her own hygiene, nutrition or housing requirements, he/she was responsible for the self-neglect. Among the participants, (43.3%) agreed that it is neglect if an older individual’s health needs were not met or not adequately met. While (43.5%) agreed that it is neglect if the older people live in homes which have unsuitable conditions (Table 3).

The overwhelming majority (99.3%) agreed that İt is abuse if an older person is exposed to violence such as beating, slapping, kicking, biting and throwing of objects in their presence, or if they are exposed to shouting, insults or ridicule. (99.8%) of the participants agreed that it is abuse if the older people money and goods are stolen, (96.7%) agreed that placing the older people in a nursing house is neglect (Table 3).

The majority, (73.7%) agreed that older women are exposed to abuse and neglect more than men, (81–84%) agreed that those who have mental and physical disabilities are exposed to abuse and neglect more often. Meanwhile, (70.5%) disagreed that older individuals who live in a large family are exposed to abuse and neglect more often, while (52.8%) of participants agreed that elder abuse and neglect is seen more often in families whose socioeconomic and cultural status is low. In addition, (81.9%) agreed that individuals who have negative feelings or thoughts toward, or bad experiences with, the older people are more likely to neglect and abuse them, while (78.4%) agreed that İndividuals who think of looking after the older people as a burden are more likely to neglect and abuse them (Table 4).

**Table 4:** Knowledge regarding possible causes of elder abuse

		age		Gender		education		living		caregiver		disability		Total	
		< 30 yrs	30+ yrs	male	Female	average	higher	large family	nuclear family	yes	no	No disability	disability		
v23 older women are exposed to abuse and neglect more	Agree	77.6	71.7	74.0	73.5	73.4	73.9	71.4	75.5	71.0	74.5	78.6	67.7	317	73.7
	Disagree	9.5	8.8	8.8	9.3	7.9	9.9	10.8	7.8	10.8	8.6	7.1	12.3	39	9.1
	neutral	12.9	19.4	17.2	17.2	18.6	16.2	17.8	16.7	18.3	16.9	14.3	20.0	74	17.2
v24 older people who have mental disability are exposed to abuse and neglect more often	Agree	83.7	84.8	86.5	82.3	80.8	87.0	81.1	86.9	79.6	85.8	85.7	76.9	363	84.4
	Disagree	5.4	3.9	4.7	4.2	6.2	3.2	6.5	2.9	7.5	3.6	3.6	9.2	19	4.4
	neutral	10.9	11.3	8.8	13.5	13.0	9.9	12.4	10.2	12.9	10.7	10.7	13.8	48	11.2
v25 older people who have physical disability, have had a stroke, or are handicapped or bedridden are exposed to abuse and neglect	Agree	80.3	82.0	84.2	78.6	79.7	82.6	78.4	83.7	76.3	82.8	85.7	72.3	350	81.4
	Disagree	8.8	6.0	6.5	7.4	7.3	6.7	8.1	6.1	10.8	5.9	3.6	13.8	30	7.0
	neutral	10.9	12.0	9.3	14.0	13.0	10.7	13.5	10.2	12.9	11.3	10.7	13.8	50	11.6
v26 older people who live in a large family are exposed to abuse and neglect more often	Agree	9.5	15.5	10.2	16.7_b_	15.3	12.3	13.5	13.5	11.8	13.9	10.7	12.3	58	13.5
	Disagree	72.1	69.6	73.5	67.4	66.7	73.1	69.7	71.0	73.1	69.7	75.0	72.3	303	70.5
	neutral	18.4	14.8	16.3	15.8	18.1	14.6	16.8	15.5	15.1	16.3	14.3	15.4	69	16.0
v27 elder abuse and neglect is seen more often in families whose socioeconomic and cultural status is low	Agree	54.4	51.9	48.4	57.2	55.9	50.6	50.3	54.7	50.5	53.4	35.7	56.9	227	52.8
	Disagree	25.2	29.7	31.6	24.7	29.4	27.3	31.4	25.7	32.3	27.0	46.4	26.2	121	28.1
	neutral	20.4	18.4	20.0	18.1	14.7	22.1	18.4	19.6	17.2	19.6	17.9	16.9	82	19.1
v28 Individuals who have negative feelings toward, thoughts about and bad experiences with the older people are more likely to neglect and abuse them	Agree	81.0	82.3	79.1	84.7	82.5	81.4	81.1	82.4	83.9	81.3	82.1	84.6	352	81.9
	Disagree	8.8	8.8	11.2	6.5	10.7	7.5	9.7	8.2	7.5	9.2	10.7	6.2	38	8.8
	neutral	10.2	8.8	9.8	8.8	6.8	11.1	9.2	9.4	8.6	9.5	7.1	9.2	40	9.3
v29 İndividuals who think looking after the older people as a burden are more likely to neglect and abuse them	Agree	75.5	79.9	76.3	80.5	78.5	78.3	76.8	79.6	78.5	78.3	78.6	78.5	337	78.4
	Disagree	13.6	10.2	13.0	9.8	13.0	10.3	12.4	10.6	11.8	11.3	14.3	10.8	49	11.4
	neutral	10.9	9.9	10.7	9.8	8.5	11.5	10.8	9.8	9.7	10.4	7.1	10.8	44	10.2
Knowledge	Min-max	0–100	14.29–100	0–100	0–100	0–100	14.29–100	0–100	0–100	14.29–85.71	0–100	28.57–85.71	14.29–85.71	0–100	
	Mean	65.69	67.14	66.45	66.84	66.1	67.02	65.02	67.87	65.75	66.89	65.31	65.93	66.64	
	Median	71.43	71.43	71.43	71.43	71.43	71.43	71.43	71.43	71.43	71.43	71.43	71.43		
	SD	22.6	20.16	21.85	20.18	21.36	20.79	22.78	19.53	20.97	21.04	17.57	22.4	21.01	

a and b are significantly different at *p* < .05 in the two-sided test of equality for column means. Otherwise no significant difference is present Tests are adjusted for all pairwise comparisons within a row using the Bonferroni correction

## Discussion

As clarified before, elder abuse is a problem that needs additional focus and better education for the people taking care of geriatrics. Elder abuse is a problem occurring in both rich and poor countries and at all levels of society [2]. This problem could aggravate as the speed of population aging worldwide is likely to lead to an increase in its incidence and its prevalence [2].

In Saudi Arabia (72%) of the whole population are between the ages of 15 to 64 years old [9]. This will lead to a significant increase to the number of the elderlies in the next decades.

This study is an attempt to explore the knowledge and attitudes of adult visitors to PHCCs in Al Qatif towards elder abuse. The sample consisted of mainly the young and middle-aged, providing a wide spectrum of ages. The percentage of females and males was almost the same, and is roughly representative of the population of Saudi Arabia. No similar previous study has been conducted in Saudi Arabia as far as we know.

When participants’ family types, environments, and whether or not they lived with an older person were evaluated, it was seen that most of them, (57%), lived in small families with children. Only (43%) of participants lived with an older individual. It is known that there are large demographic differences between developing and developed countries in living situations. According to WHO, Saudi Arabia is considered a semi-developed country. In developed countries, most of the older people live in urban areas, While in developing countries, most of them live in rural areas. If current trends continue, there will be a much larger older population in the future [6]. According to the literature, older individuals who live in large families and have attractive founds are more likely exposed to abuse [6]. Most participants in our research did not agree that older individuals who live in a large family are more likely exposed to abuse and neglect. Therefore, this conflict needs to be addressed in future research.

In previous research, it was shown that (62%) of poor older individuals are exposed to abuse, while only (6%) of the older people from wealthy households are abused [10].

Our study showed that (52.8%) of the participants believed that elder abuse is more common in families with low socio-economic status. A previous study supports the opinions of our study participants. It showed that (62%) of poor older individuals are exposed to abuse, while only (6%) of the older people from wealthy households are abused [10].

A sizable majority (77%) of participants disagreed that the older people would be less likely to suffer violence if they were in a nursing home with their peers, rather than living with their caregivers. There were different findings in this issue from the previous literature. A study suggests that between (4%) and (6%) of older persons have experienced some form of abuse in the home. Also, older persons are at risk of abuse in institutions such as hospitals, nursing homes and other long-term care facilities, but no large-scale measuring studies are available [2]. There are limited numbers of studies about this topic in Saudi Arabia.

The results showed that most participants (90.5%) considered manhandling of older parents/in-laws to be violence/abuse, and (87.7%) considered touching intimate body areas of the older people without their consent to be sexual abuse. A previous research supports the participant opinion [11]. In this research sexual abuse was defined as touching, fondling, intercourse, or any other sexual activity with an older adult when the older adult was unable to understand, unwilling to consent, threatened, or physically forced [11].

Eighty-one percent of participants agreed that caregivers who have negative feelings or thoughts toward or have had bad experiences with the older people are more likely to neglect and abuse them. Also, (78.4%) of participants agreed that caregivers who think of looking after the older people as a burden are more likely to neglect and abuse the. In addition, (99.3%) agreed that it is abuse if the older people are exposed to shouting, insults and ridicule. The opinions of the participants of the survey correspond with the findings from a previous research regarding the features of individuals who practice elder abuse [6]. These individuals have personality issues (such as not being able to control emotions and behaviour), financial or medical problems, marital conflict, unemployment, alcohol or drug addiction, and perceiving violence as a solution [6].

An almost unanimous majority (99.8%) of participants agreed that it is abuse if an older person’s money and goods are stolen, whether obtained by force or deception. Also, (90.7%) agreed that borrowing money from older parents and not returning it is violence. Abuse is the physical, psychological or financial mistreatment of an older person by an individual, who has a relationship with them. As mentioned before financial abuse is considered one type of elder abuse [2]. In a study regarding 204 participants who are 65 years and over in Turkey, it was found that (2.5%) of them met financial abuse [6].

Seventy-five percent of participants agreed that the older people are less likely to be exposed to violence if they understand the problems of their children. A study carried out in the US on the relationship between mothers/fathers and their children is mentioned and evaluated. Young mothers and fathers are especially likely to consult their family regarding raising their children, because they accept their family’s expertise on child education [6]. The study also found that care for the older people is often an obligatory duty and not voluntary. It is stated that owing to the social structure of home care conditions, the primary responsibility for older people care is met by adult children, but abuse and neglect are also engaged in by spouses, other relatives and health care staff [6].

Fifty-five percent of participants agreed that placement of the older people in a nursing home was neglect, while (27%) disagreed, and the remaining (18%) were neutral regarding this matter. Similar findings was identified in a previous study in Turkey. In that study, most of the participants thought that placing the older people in nursing home is an elderly neglect [6]. Those thoughts could be originated from an Islamic perspective or a cultural one. Both Turkey and Saudi Arabia share the same Islamic values and share a similar culture, however no study conducted was done in Saudi Arabia regarding this point.

There was nearly unanimous (99%) agreement that violence such as beating, slapping, kicking, biting or throwing objects is abuse. This agrees with the American Psychological Association consideration of physical abuse as one of the types of elder abuse [2].

Most of participants agreed with the statement that “Using swear words is considered to be elder abuse because it is uncommon in the culture”. A previous study in North America observed that women and older people reported suffering verbal abuse more frequently [12]. We could not find a study in Saudi Arabia regarding this point.

Nearly all participants (96%) agreed that abandonment of the older people is considered neglect. The opinions of participants match the opinions of participants in another study in Turkey. In that study, most of the participants think that abandoning the older people is elderly neglect [6].

(43.3%) of participants agreed that “İt is neglect if the elderly’s health needs aren’t met or are not met in a timely fashion, while (43%) of participants agreed that it is neglect if the older people live in homes which have unsuitable conditions. A previous study supports matches participant opinions. It mentioned that both caregivers/families and the older people need support related to care. When this support was not timely or adequate, elder abuse and neglect have occurred [6].

Almost all of our participants (96%) agreed that it is abuse to act as if older people relatives don’t exist, imprison them in their room/home and exclude them from society. A previous study in Turkey goes with the participant thought. The study mentions that older people between 70 and 75 years, woman who are divorced, living alone, being isolated and having some chronic diseases are exposed to abuse mostly [6].

Most of the participants disagreed with the statement that “If older individuals can’t meet their own hygiene, nutrition and housing requirements, it means that they are neglecting themselves”. In previous researches it was found that it is very common for the older people to have decreased physical and mental ability and to need care [6].

In our study, most of the participants (98.1%) agreed with the statement that it is their individual responsibility to report elder abuse and neglect if they witness any, and (98.1%) of them also considered elder abuse and neglect to be a criminal act and believed that they have a responsibility to prevent it. Similarly, in a study in Turkey (89%) of participants believed that elder abuse and neglect is a social problem and they have professional and individual responsibilities to prevent it [6].

The majority of our participants, (73.7%), believed that older women are more exposed to abuse and neglect than men, and there was no significant difference between female and male participants in their opinion regarding this. A study in Thailand found that women were approximately five times more likely to have been abused than men [1], The results of our survey matches the study in Thailand.

Most of our participants (84.4%) agreed that older individuals who have mental disability are exposed to abuse and neglect more often. Similarly, (81,4%) of participants agreed that older individuals who have physical disabilities or are bedridden are more often exposed to abuse and neglect. A recent study found that individuals with less functional and cognitive impairment might present more disruptive behaviour and greater interaction with the caregiver, which could be associated with a higher risk of abuse [12]. Similarly, the study found that when the caregiver reported aggressive behaviour inflicted by the care recipient, the risk of all types of abuse increased; nevertheless, such aggressive situations occurred less frequently when the dependency was total [12]. The findings in our survey go in line with the study results.

Our study is the first research in Saudi Arabia that identified the attitudes and awareness toward elder abuse in the Saudi community, the participant in our study responded positive when asked if they had a responsibility to prevent and report elder abuse. One of the possible reasons of this view among those in our study group might be the rapid cultural and geodemographic changes in Saudi Arabia due to people moving from rural to urban area, which lead to enhancements in their education, including education about elder abuse. A further research is needed to examine this conclusion.

As an intervention and prevention measurements to reduce the incidence of elder abuse we could suggest that we include the elderlies in the community festivals and events so that they don’t become isolated from the social life, we could also use a special annual day for the elderlies were both the younger generation and the elders can participate in. As for education the public, school programs can be used to educate the young from early life about this matter also using campaigns] [13].

Our research was focused on the attitude and the knowledge of the age group from 18 to 59 years old we could suggest a specific research for the elderlies and their own attitude regarding this matter. Also, one of the findings of our research was that most of the participants believed that females get abused more than the males. This assertion needs to be investigated to find the motives.

### Limitations

There were some limitations in the study. First we didn’t include the older population in the samples and there weren’t any previous studies about the attitude of the older Saudi population about this matter. This might be due to the difficulty to collect a large sample from the older population who visits the PHCCs, in Saudi Arabia people who are older than 65 years old represents only 3.2% of the whole population [14].

The second limitation was that we only conducted this study in the eastern province of Saudi Arabia. This was due to the large size of the kingdom of Saudi Arabia which will take a large amount of resources and time to include other regions.

## Conclusion

The current study was carried out with Saudi attendees at a public care facility in Saudi Arabia. It is the first study about public attitudes toward elder abuse and neglect.

In this study, participants agreed that they had responsibility to take action to address older people neglect and abuse that they observed. This positive attitude will help and support older people who are facing such abuse. However, this not enough to protect older people from abuse. The study indicates that people in Eastern Region of Saudi Arabia require more education about the various types of abuse and negligence. They also need counselling regarding the needs of older people and how to identify the signs and symptoms of abuse. In addition, they need to be educated on how to prevent and report elder abuse. Ensuring all of these requirements are met will help preventing elder abuse.

## Data Availability

The datasets used or analysed during the current study are available from the corresponding author on reasonable request.

## Abbreviations

PHCCs: Primary health care centers
SPSS: Statistical product and service solutions
WHO: World health organization
